# Neisseria Gonorrhoae and their antimicrobial susceptibility patterns among symptomatic patients from Gondar town, north West Ethiopia

**DOI:** 10.1186/s13756-018-0376-3

**Published:** 2018-07-17

**Authors:** Addisu Gize Yeshanew, Rozina Ambachew Geremew

**Affiliations:** Department of Microbiology, St. Paul’s Hospital Millennium Medical College, Addis Ababa, Ethiopia

**Keywords:** *N. Gonorrhoeae*, Antimicrobial sensitivity test

## Abstract

**Background:**

*Neisseria gonorrhoeae*, the causative agent of gonorrhoea, is a Gram negative, coffee-bean shaped facultative intracellular diplococcus bacterium, the classical sexually transmitted bacteria. Nowadays, *N. gonorrhoeae* has developed high-level resistance to all traditional antimicrobials used for the treatment of gonorrhoea and makes the treatment complicated. So, the aim of this study was to determine magnitude and antimicrobial resistance patterns of *N. gonorrhoeae.*

**Methods:**

A cross sectional study was conducted between April and August 2016 among STI clinic clients in Gondar town hospitals and health centers. Urethral swab and cervical swab specimens were inoculated to Thayer Martin media (OXOID, UK) plates. Observation of Gram-negative intracellular diplococcic was a presumptive diagnosis of gonococcal infection. Finally, antimicrobial susceptibility was assessed by using a modified Kirby-Bauer disk diffusion test, with results indicating susceptible, intermediate or resistant. Data were entered and analyzed using SPSS version 20.

**Results:**

The overall prevalence of laboratory confirmed N*. gonorrhoeae* was 25(20.8%). The isolated N. *gonorrhoeae* was resistant 100% to tetracycline, penicillin and 80% percent was multidrug resistant.

**Conclusion:**

Prevalence and drug resistance of *N*. *gonorrhoeae* were high in the study area. A large study is needed in order to know the magnitude in the community as well as to increase awareness of both regional health bureaus and the Ministry of Health about the treatment guide-lines.

## Background

*Neisseria gonorrhoeae* is a Gram negative, coffee-bean shaped intracellular diplococcus bacterium responsible for gonorrhoea which is one of the classical sexually transmitted infections (STIs) [1]. The causative organism is highly adapted to the genital tract and often causing asymptomatic and undetected infection [2]. Gonorrhoea is acquired by unsafe sex practices and also can be transmitted from mother to child during delivery [1], which has medically significant implications if not treated early. Untreated gonorrhoea can lead infertility, inflammation leading to acute and chronic abdominal pain in women, ectopic pregnancy and maternal death, first trimester abortion, severe neonatal eye infection that lead to blindness, a fivefold increase in HIV transmission, disseminated gonococcal infection which results in localized septic arthritis, endocarditis, and meningitis [1, 3, 4]. Therefore, accurate efforts should be made to offer routine endocervical cultures for *N. gonorrhoeae* to all women who have risk behavior of STIs to prevent this infection [5]. Gonorrhoea treatment is complicated by *N.gonorrhoeae’s* ability to develop antimicrobial resistance [6]. Since the introduction of antimicrobials to treat *N. gonorrhoeae* infection 70–80 years ago, *N. gonorrhoeae* has progressively developed resistance to most antimicrobial previously recommended for treatment, including sulfonamides, penicillin, tetracycline, and fluoroquinolone antibiotics [7]. In European Surveillance of combined antimicrobial susceptibility data, the resistance was found to be high to ciprofloxacin (30.9%), penicillin (21.3%) and tetracycline (59.8%), and Neisseria gonorrhoeae strain (H041) that is highly resistant to the extended-spectrum cephalosporin (ESC) ceftriaxone [8, 9].

In many parts of the developing world, the absence of etiologic diagnostic capacity due to constraints imposed by cost, lack of equipment or trained personnel, and time management has forced health care providers to rely on a syndrome based approach to STI management, because many STIs have common symptoms or are asymptomatic and therefore undetected and untreated [10]. Therefore, this study primarily aimed to determine the prevalence *N. gonorrhoeae* and their antimicrobial susceptibility pattern among STI clinics attendants at Gondar town, North West of Ethiopia.

## Materials and methods

A cross-sectional study was conducted from April 1 to August 30, 2016 in Gondar town hospitals and health centers, Gondar town, Northwest Ethiopia. The Town is 725 km away from Addis Ababa, Ethiopia, located in the North Gondar Zone of the Amhara Region. Based on figures from 2011 Central Statistical Agency (CSA) of Ethiopia, Gondar town has a total population of 207,044, of whom 98,120 are men and 108,924 women. There are two hospital (1 governmental and 1 private) and 5 health centers in the town.

Patients who visited Gondar town hospitals and health centers during the study period serve as the source population, whereas, all STI suspected patients attending in the STI clinic were included as the case of the study. However, patients under antibiotic treatment during 7 days before the enrolment and female patients who were on menstruation at the time of examination were excluded from the study. Descriptive statistics, including socio-demographics factors and known risk factors for STI were performed.

### Sample size and sampling technique

Sample size was calculated based on the single population proportion formula considering the prevalence rate of 8.5% from the previous study conducted in Bahirdar, Northwest Ethiopia. Expected margin of error (d) was 0.05 and confidence interval (z) was 95%. One hundred twenty consecutive consenting patients, who attended Gondar town hospitals and health centers STI clinic, with one or more of the complaints as stated by WHO in its syndrome approach for the diagnosis of STI were included [5].

### Data collection procedure

#### Socio-demographic characteristics

After taking written informed consent from each study participant, a semi-structured questionnaire was used to collect socio-demographic and clinical data needed in this study. Data were collected by trained nurses.

### Sample collection

#### Urethral and cervical specimen

External inspections of the genital area were made, and the characteristics of any local changes such as; erythema, abrasions, ulceration, urethral discharge and vaginal discharge were noted, including color, amount, odor and consistency. In the absence of visible urethral discharge, the patient was asked to milk the urethra. Specimens of discharge were obtained at or just within the urethral meatus for male and cervical swabs were taken for female. Two swabs were taken; one for Gram staining and the other were put on Amies transport media for *N*. *gonorrhoeae* culture at Gondar University Hospital laboratory. This laboratory, according to Ethiopian National Accreditation Office (ENAO), which established the Stepwise Laboratory (Quality) Improvement Process towards Accreditation (SLIPTA), has got 3 stars (75–84%) ranked from 5 stars (≥95%). ENAO gives recognition based on progress towards meeting requirements set by international standards and on laboratory performance during the 12 months preceding the SLIPTA audit, relying on complete and accurate data, to improve quality of public health laboratories in developing countries to achieve ISO 15189 standards.

### Laboratory method

#### Isolation and identification of N. Gonorrhoeae

Identification was made by observing polymorphonuclear leucocytes (PMNLs) with Gram-negative intracellular diplococci as a presumptive diagnosis of gonococcal infection. Specimens inoculated directly into the modified Thayer Martin medium (MTM) (OXOID, UK) plates contained vancomycin, colistin, nistatin and trimetoprin and incubated at 36.5 °C for 24–48 h. Then isolates was identified as *N*. *gonorrhoeae* on the basis of colony morphology, Gram staining, oxidase test, and carbohydrate utilization test [11]. *N. gonorrhoeae* is observed as Gram negative diplococci when Gram stained, positive for oxidase, ferments only glucose, and is resistant to colisin [12].

### Antibiotic susceptibility testing

From a pure culture, 3–5 selected colonies of bacteria were transferred to a tube with a straight wire and prepared in a suspension of 2.5 ml normal saline and incubated at 36.5 °C until the turbidity of the suspension become adjusted to a McFarland 0.5. A sterile swab was used to distribute the bacteria evenly over the entire surface of MTM agar. The susceptibility to the following antimicrobial agents (OXOID, UK) were assessed: penicillin (P 10 IU), tetracycline (TE 30 μg), ciprofloxacin (CIP 5 μg), ceftriaxone (CRO 30 μg), Cefotaxime (30 μg), Cefoxitin (FOX 30 μg), Clindmycin (CLI 2 μg). The criteria used to select the antimicrobial agents tested were based on their availability and frequent prescriptions for the management of gonococcal infection and the national list of medicines by Food, Medicine and Health- Care Administration and Control Authority (FMHACA) Ethiopia in 2010 to treat infections, and the syndromic management package for the management of sexually transmitted infections guideline from the Federal Ministry of Health.

Finally, antimicrobial susceptibility testing was performed for all isolates according to the criteria of Clinical and Laboratory Standard Institute (CLSI) by the modified Kirby-Bauer disk diffusion method on Thayer Martin medium plates, results as to susceptible or resistant were based on the M100-S24 Performance Standards for Antimicrobial Susceptibility Testing [12].

### Data quality control

The questionnaire was pre-tested on 15 outpatient department patients for comprehensiveness, effectiveness, reliability and validity. Training was given for data collectors on data collection procedures with support from an investigator, venereologist and gynecologist. Culture media sterility was ensured by incubating 5% of each batch of the prepared media at 37^o^c for 24 h. Performance of prepared media was also checked by inoculating with control strains *N. gonorrhoeae ATCCTM* 49226 (10) [13].

### Data analysis

Data were entered and analyzed using SPSS version 20. Descriptive statistics was applied to determine the distribution of the socio-demographic and clinical characteristics.

### Ethical consideration

The proposed study was approved by the research and ethics committee of the School of Biomedical and Laboratory Sciences of the University of Gondar. Official permission was obtained from each of the health facilities. The aim and details of the study was explained to each study participant before obtaining their written consent for specimen collection. Confidentiality was maintained at all levels of the study by using codes rather than names of the study participants. The results of all positive individuals were reported to the respective health facilities for better management of the patients.

## Results

### Socio-demographic characteristics

A total of 120 patients, 21 (17.5%) males and 99 (82.5%) females, were enrolled in the study**.** The mean ± SD age of the study participants was 27.8 ± 7.2 years. The majority (66.6%) of the study participants were in the age group below 30 years old. Fifty eight (48.3%) were married. The majority of the patients 74 (61.7%) were literate. Regarding their occupation, house wives numbered 26(21.7%), and in terms of economic status, 37 (30.8%) participants reported not to receiving income of their own (Table 1).

**Table 1:** Prevalence of *N. gonorrhoeae* described by socio-demographic characteristics and known risk factors for STI, Gondar town, North West Ethiopia from April–August 2016

Characteristics	Total N (%), *n* = 120	*N. gonorrhoeae* + ve (%),*n* = 25	*N. gonorrhoeae* - ve (%), *n* = 95
Sex			
Male	21(17.5)	6 (29)	15 (71)
Female	99(82.5)	19(19)	80(81)
Age			
15–29	80(66.7)	17(21)	63(79)
30–45	40(33,3)	8(20)	32(80)
Marital status			
Single	62(51.7)	14(23)	48(77)
Married	58(48.3)	11(19)	47(81)
Educational status			
Illiterate	46(38.3)	9(20)	37(80)
Literate	74(61.7)	16(22)	58(78)
Occupation			
Government employed	31(25.8)	7(23)	24(77)
House wife	26(21.7)	3(11)	23(89)
CSW	11(9.2)	2(18)	9(82)
No	23(19.2)	5(22)	18(78)
Self-employed	29(24.1)	8(18)	21(72)
Income			
No	37(30.8)	7(19)	30(81)
< 800	25(20.8)	7(18)	18(72)
801–1600	31(25.8)	5(16)	26(84)
> 1600	27(22.5)	6(22)	21(78)
Knowledge about STI			
No	54(45)	10(19)	44(81)
Yes	66(55)	15(23)	51(77)
Alcohol use			
No	39(32.5)	5(13)	34(87)
Yes	81(67.5)	20(25)	61(75)
Khat chewing			
No	102(86)	21(21)	81(79)
Yes	18(14)	4(28)	13(72)
Smoking			
No	111(93)	22(20)	89(80)
Yes	9(7.5)	3(33)	6(67)
History of abortion			
No	55(55.5)	12(22)	43(78)
Yes	44(44.5)	7(15)	37(84)
Sexual partner			
One partner	38(31.6)	7(18)	31(82)
More than one partner	82(68.3)	18(22)	64(78)
Condom use			
Never	69(57.5)	13(19)	56(81)
S/Rarely	38(32)	9(24)	29(76)
Always	13(10.5)	3(15)	11(85)
Males sexual contact with prostitute women			
No	16(13)	1(6)	15(94)
S/Rarely	100(83)	21(21)	79(79)
Yes	5(4)	3(60)	2(40)
History of STI			
No	53(44.2)	9(17)	44(83)
Yes	67(55.8)	16(24)	51(76)
Commonly using toillet & washing materials			
No	81(67.5)	19(23)	62(77)
Yes	39(32.5)	6(15)	33(85)
Contact new person the last 3 months			
No	78(65)	16(21)	62(79)
Yes	42(35)	9(21)	33(79)
HIV status			
Positive	17(14.2)	1(6)	16(94)
Negative	103(86)	24(23)	79(77)

+ *ve* infected with N. gonorrhoeae, , *ve* non-infected with N. gonorrhoeae, *S/Rarely* sometimes or rarely, *CSW* Commercial Sex Workers, *STI* Sexual Transmitted Infection, *HIV* Human Immunodeficiency Virus

### Prevalence of N. Gonorrhoeae

The overall prevalence of laboratory confirmed *N. gonorrhoeae* pathogens among suspected attendees of Gondar town hospitals and health centers was 25(20.8%). N*. gonorrhoeae* infections were higher among females and age groups 15-29 yrs. The majority of the respondents 82(68.3%) had multiple sexual partners, and 69(57.5%) had never used condoms (Table 1).

### *.*Antimicrobial susceptibility pattern of N. Gonorrhoeae strains

The antimicrobial susceptibility pattern of all isolates of *N. gonorrhoeae* is shown in Table 2. All isolates of *N. gonorrhoeae* showed resistance to at least one antimicrobial agent. The susceptibility pattern of isolates shows there were a hundred percent non-susceptibility for tetracycline, 76% non-susceptibility and 24% intermediate resistant for penicillin. The isolated *N. gonorrhoeae* was resistant 52% to ciprofloxacin, 48% to ceftriaxone, 44% to cefoxitin, 29% to cefotaxime, and 28% to clindmycin. Most of the isolates (80%) showed multiple drug resistance and 11(44%) of the isolates showed non-susceptibility to both ciprofloxacin and ceftriaxone.

**Table 2:** Antimicrobial susceptibility pattern of *N. gonorrhoeae* isolates from STI clinic attendants in Gondar town, North West Ethiopia from April–May 2016

Antibiotic	#S (%)	# I (%)	# R (%)
Penicillin	0(0)	6(24)	19(76)
Tetracycline	0(0)	0(0)	25(100)
Ciprofloxacin	4(16)	8(32)	13(52)
Ceftriaxone	13(52)	*	12(48)
Cefotaxime	17(71)	*	7(29)
Cefoxitin	14(56)	*	11(44)
Clindmycin	18(72)	*	7(28)

# *S* number of sensitive, # *I* number of intermediate, # *R* number of resistance, * have no intermediate range

## Discussion

The overall prevalence of laboratory confirmed *N*. *gonorrhoeae* was 25(20.8%). This prevalence rate was comparable with studies from Mozambique (22.5%) [14], Egypt (26%) [15] and southwestern Nigeria (25%) [16]. However it was higher than the value reported from previous findings of studies done in Hawassa, Southern Ethiopia (5.1%) [2] as well as the Northern part of Ethiopia [17]. Again the present finding was higher than the studies conducted in other countries: for example, 11% in Mongolia [18], 6% United States [19], 1.4% in Colombia [20], 6% in India and 1% from Nigeria [21]. These discrepancies might be due to the lack of advancement in both early diagnosis and treatment in our country, which can lead to an increased number of untreated patients. A lack of differential diagnosis for resource poor settings can lead to an increased number of untreated patients and a poor partner tracing system and using a syndromic approach, especially in the study area, might be the possible causes for this high prevalence rate.

The main reason for the rapid increase of *N. gonorrhoeae* resistance rates to Penicillin, Tetracycline and Ciprofloxacin after they had been recommended for clinical use is likely their indiscriminate use. Patients tend to administer antimicrobial treatment by themselves or visit non-formal medical institutions such as unregistered private clinics. Moreover, some patients do not complete the full treatment course, increasing the chance for antimicrobial resistance to develop. For example, patients could buy antimicrobials in drug stores without a prescription. Indeed, an association between self-prescribed antimicrobial use and gonococcal AMR may be high.

Gonococcal infection was observed in 6(29%) of males and, patients having multiple sexual partners 18(22%) and previous history of STI 16(24%). This prevalence rate may be attributed to less condom usage by females and having a history of STI which leads to the infection. The same factors were shown to increase the exposure and prevalence rate of gonococcal infection in other studies as well [22, 23].

In addition, it was observed infection in both the age groups of 15–29 & 30–45 years old. The finding on the target group was also supported by other studies [24, 25].

The antimicrobial resistance patterns ranged from 7(28%) for Cefotaxime and Clindmycin up to 100% in Tetracycline. This Tetracycline resistance pattern was similar with the study done in Gambella hospital, Ethiopia [1]. The present study showed a high level of resistance to Tetracycline 25(100%) and Ciprofloxacin 13(52%) compared to the study done in North West Ethiopia (92.6, 40.9%) (15), Southern Ethiopia (55, 18%) [2] and United States (25.3, 19.2%) [4].

Most researchers are worried gonorrhoea will soon become untreatable with those antibiotics. This may be true in resource limited countries because of a lack of proper usage of antibiotics and application of antimicrobials as therapeutic agents for syndromatic treatment or may be due to the emergence of new resistant beta-lactamase producing strains over a period of time.

The resistance of Cefotaxime 7(28%) and Cefoxitin 11(44%) in the current study is also higher than the study conducted in Gambella hospital, Ethiopia [1] and Southern Ethiopia [2] but lower as compared to Tetracycline, Penicillin, Ciprofloxacin. The reason might be these drugs are expensive, not intensively used and not easily available outside the health institution, plus these drugs are newer compared to the others. Low resistance pattern of these drugs in our study may make these drugs excellent choices as first-line treatment.

## Conclusion

High prevalence of *N. gonorrhoeae* and drug resistance among symptomatic clients who attended the STI clinics in the study area was observed. This should cause alarm and should lead to a large community based study and increased awareness of both regional health bureaus and the Ministry of Health about the treatment guide-lines.

## Abbreviations

AMR: Antimicrobial Resistance
CLSI: Clinical and Laboratory Standards Institute
ESBL: Extended-spectrum β-lactamase
FDA: US Food and Drugs Administration
HIV: Human Immunodeficiency Virus
SPSS: Statistical Packages for Social Sciences.
STI: Sexually Transmitted Infections
